# Emergency medical services, treatment of cardiac arrest patients and cardiac arrest registries in Europe – Update on systems

**DOI:** 10.1016/j.resplu.2025.100960

**Published:** 2025-04-16

**Authors:** Anneli Strömsöe, Ingvild B.M. Tjelmeland, Siobhan Masterson

**Affiliations:** a School of Health and Welfare, Dalarna University S-79188 Falun, Sweden; b Centre for Clinical Research Dalarna, Uppsala University S-79182 Falun, Sweden; c Department of Prehospital Care, Region of Dalarna, S-79129 Falun, Sweden; d Division of Prehospital Services, Oslo University Hospital, Norway; e HSE National Ambulance Service, Ireland

**Keywords:** Epidemiology, Emergency Medical Systems, Out-of-hospital cardiac arrest

## Abstract

**Introduction:**

Incidence and survival rates following cardiac arrest vary significantly across Europe. While several studies have sought to address the knowledge gap in the epidemiology of out-of-hospital cardiac arrest (OHCA), they have not successfully identified the reasons behind these disparities. This study aims to provide an updated overview of European Emergency Medical Systems (EMS).

**Methods:**

A questionnaire consisting of 35 main questions was used. The survey encompassed topics related to ambulance and dispatch characteristics, on-scene cardiac arrest management, as well as the availability and scope of datasets in cardiac arrest registries.

**Results:**

Survey responses were received from 27 European countries. While there were differences in the proportion of staff with advanced life support skills between countries, these staff were almost invariably dispatched in the event of a cardiac arrest call. First responder systems were available in only 17 countries. There were huge differences in ambulance control models, with the number of dispatch centres ranging from 0.4 to 42.2 per million population. Nine countries reported having out-of-hospital registries of prehospital cardiac arrest with national coverage while only three countries had registries of in-hospital cardiac arrest with full coverage.

**Conclusions:**

There are differences in EMS structures and the management of OHCA across Europe. Understanding these discrepancies is essential for improving OHCA outcomes and fostering greater uniformity in emergency response protocols throughout the region. Although there has been an increase in the population covered by a cardiac arrest registry, there is still a need to expand registry coverage, especially for registries of in-hospital cardiac arrest.

## Background

Out-of-hospital cardiac arrest (OHCA) is one of the most common causes of unexpected death from a global perspective. The prognosis for patients who sustain cardiac arrest differs between countries, but the common denominator is that survival is still low.^1^, ^2^ Common factors among patients with OHCA are that cardiopulmonary resuscitation (CPR) is not initiated after the collapse, the event was not witnessed, and the time to CPR initiation is too long to achieve a meaningful resuscitation outcome.^2^

Previous studies of ambulance organisational structure from the last 5 years are limited. Gaining insight into the diverse organisational models of EMS is essential for understanding their performance and impact on a global scale. This perspective is particularly valuable for countries in the early stages of EMS development, as it provides a reference for system organisation in other nations. Additionally, it enables European countries to assess and compare their EMS structures, fostering opportunities for mutual learning and adaptation. Professions such as paramedics, emergency medical technicians (EMT), nurses, and physicians, make up the staff in European EMS systems, but varies between countries.^3^ The EMS organisation usually consists of ground emergency medical service (GEMS) and helicopter emergency medical service (HEMS).^4^ It forms the basis for caring for acute and non-acute medical conditions in the prehospital setting. A previous study described that, in addition to ambulances and helicopters, motorcycles are also used.^5^ From the last five years, only two articles describing EMS systems were identified.^6^, ^7^

This study aims to provide an updated overview of EMS in Europe and thereby describe differences and similarities that might help explain some of the differences in the data published on prehospital emergencies, treatments and outcomes.

## Methods

A structured questionnaire from 2019,^8^ developed through a review of published literature on previous international ambulance surveys,^9^, ^10^, ^11^, ^12^, ^13^ was repeated with some small changes. Five questions were omitted as they were not within the scope of the paper, and three questions were omitted due as all regions reported similar answers in 2019. The questionnaire contained 35 main questions, with follow-up to some questions, and a matrix of available answers for two questions regarding cardiac arrest registry data (Additional file 1; EMS survey). It was designed to investigate five categories:

1. Country and EMS baseline characteristics
2. Ambulance Service characteristics
3. Dispatch characteristics
4. On-Scene Management of Out-of-Hospital Cardiac Arrest by the EMS
5. Cardiac Arrest Registries

The questionnaire was shared with participants using the online tool Nettskjema, licensed to the University of Oslo. Data was stored and analysed in an approved area at Oslo University Hospital.

The survey was distributed between December 2024 and January 2025. The European Resuscitation Council (ERC) has established a network of resuscitation registries throughout Europe, the European Registry of Cardiac Arrest (EuReCa)^14^ and national coordinators of EuReCa were invited to participate in this survey (*n* = 31). In addition, the ERC network was used to identify contacts in countries not participating in the EuReCa studies. A total of 37 invitations were sent out to 32 countries, and we had responses from 27 (84%) countries. Two reminders were sent out via email and text message.

Results were returned to all participants, including a request to confirm that the results related to national rather than local EMS organisations. Three countries repeated the survey and confirmed the new results. After all the data had been merged into a result section, the tables were again shared with the participants to validate the results. Results are presented as frequencies and proportions.

### Ethical approval

The information in this study consists of publicly available, non-personal data, and no ethical approval for collection and analysis was needed. An approval was obtained from the data protection officer at Oslo University Hospital for storage and analysis of data.

## Results

### Country information and baseline characteristics

**Fig. 1:**
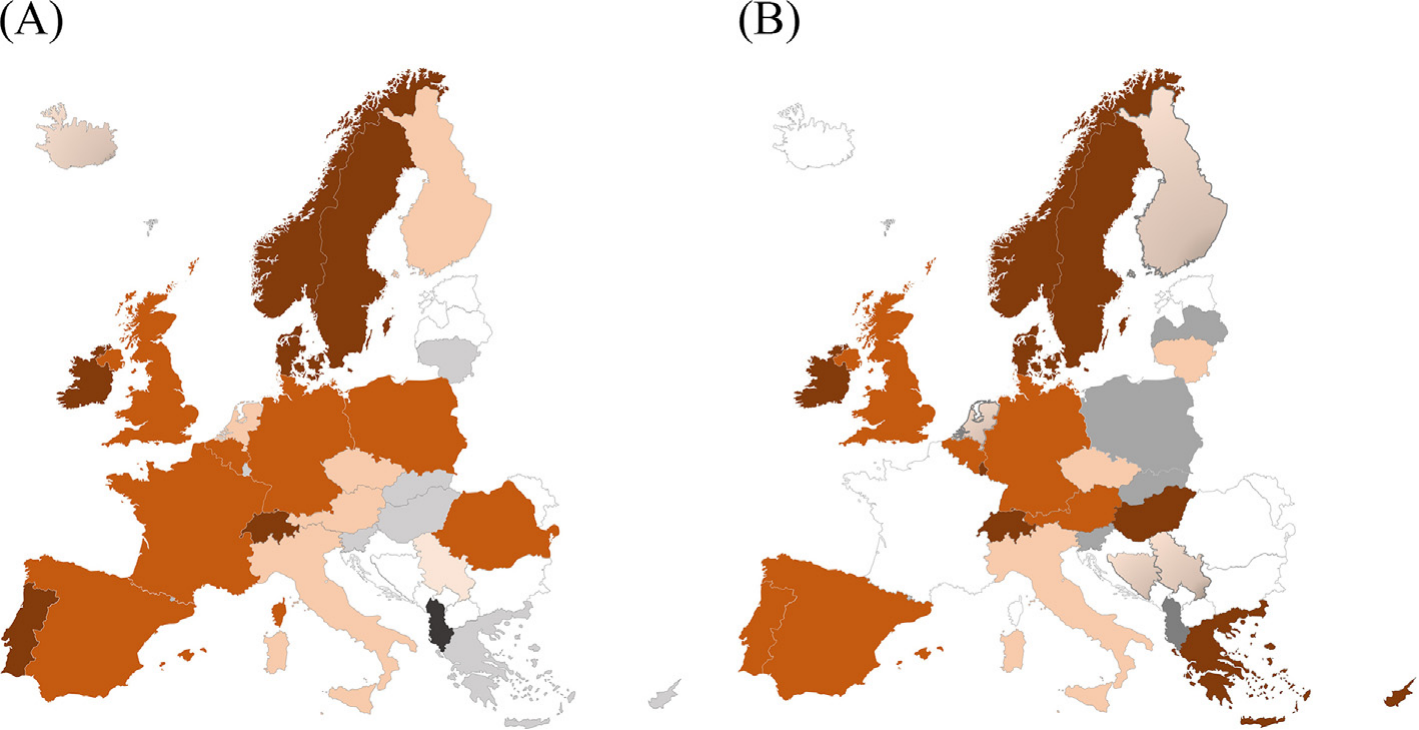
A and B: Registries reported in 2019 in A and 2024 in figure B. Dark orange is national registries covering all of the country, orange is national registries covering parts of the country. Light orange is several local registries and light orange with dark shadow is one local registry. Grey is no registry and black is unknown. Countries not participating are left white. (For interpretation of the references to colour in this figure legend, the reader is referred to the web version of this article.). (For interpretation of the references to colour in this figure legend, the reader is referred to the web version of this article.)

Responses were collected from 27 European countries. Two new countries joined the survey (Latvia and Bosnia Herzegovina) and two countries in the previous survey did not participate (Romania and France) Fig. 1. Survey responses covered a population of over 443 million people with population density ranging from 14 to 476 people per km^2^. As can be seen in Supplementary Table 1, publicly funded EMS services were available in all countries, with seven respondents reporting a mix of public and private funding. Specialised cardiac arrest centres were available in all areas in only five countries, with a further four countries reported as not having specialised centres at all. Only one country reported that the median response time for rural areas was less than ten minutes (i.e. the Netherlands). Ten countries reported a median response time of less than ten minutes in urban areas. It is noted that there was substantial variation in the time stamps used in different countries to calculate response time, and no clear pattern was observable in the time stamps used (Supplementary table 2).

### Ambulance service characteristics

As shown in Supplementary table 3, the majority (*n* = 16) of EMS personnel were reported as paramedics or Emergency Medical Technicians (EMTs). While all personnel were Advanced Life Support (ALS) trained (i.e. at the European Resuscitation Council ALS level or similar) in 11 countries, and some were ALS trained in the remaining 16 countries, in the vast majority of countries (*n* = 21), personnel on either the first or second ambulance were reported as always being ALS trained. Non-physician ambulance personnel were allowed to perform ALS procedures in the absence of a physician in the vast majority of countries, including securing airways with supraglottic or endotracheal tubes.

A Helicopter EMS (HEMS) was available in 22 countries. However, only Denmark, Norway, Switzerland and the Netherlands reported having 24/7 HEMS availability in all areas. Twelve countries operated motorcycle response units (MRUs) in some areas, with only half of these MRUs being operated by ALS-trained staff.

In 17 countries there were established first responder systems (where volunteers were alerted to OHCA by the dispatch centre) in some or all areas. Countries that were reported not to have first responder systems were Albania, Belgium, Bosnia and Herzegovina Cyprus, Finland, Latvia, Lithuania, Portugal, Serbia and Slovakia. In ten countries, volunteers were reported to staff ambulances in some areas (Belgium, Czech Republic, Germany, Spain, Luxembourg, Portugal, Poland, Hungary and Italy) or all areas (Austria). In most countries in all areas, equipment and expertise for life-threatening trauma (life, limb) was available in all emergency ambulances. In six countries this level of equipment and expertise was available only in some areas (Bosnia Herzegovina, Greece, Italy, Portugal, Serbia and Spain).

### Dispatch characteristics

The number of dispatch centres per million population ranged from 42.2 in Bosnia Herzegovina to 0.4 in Ireland (Supplementary table 4). Dispatch centres were fully part of the EMS in 16 countries, eight countries had some dispatch centres as part of the EMS, while dispatch centres were separate from EMS in three countries (Finland, Slovakia and Slovenia). Except Serbia, standardised dispatch protocols were used in all areas (*n* = 19) or some areas (*n* = 7). Every country offered dispatch-assisted Cardiopulmonary Resuscitation (DA-CPR) in some or all areas, but a standardised DA-CPR protocol was not reported to be in use in Serbia. Ten countries offered compression-only instructions while the remaining countries offered situation dependent or ‘full’ CPR instructions (no data available for Austria or Germany).

### On-scene management of out-of-hospital cardiac arrest by emergency medical services

Mechanical CPR was used in 24 countries (Supplementary table 5). In Belgium, mechanical CPR is not used by ambulance personnel, but it is used during transport. When compared to the last survey, there was more use of mechanical CPR in six countries (Czech Republic, Greece, Hungary, Luxembourg, Portugal and the UK). Transport with ongoing CPR was not performed in three countries (Albania, Bosnia Herzegovina and Serbia). When compared to the last survey, transport with ongoing CPR was more common in 21 countries. Defibrillators were reported as available in vehicles dispatched for cardiac arrest in every country. Prehospital thrombolysis was reported as being available in 15 countries (no availability in ten countries, no data reported for Poland and Switzerland), and was more commonly available in four countries when compared with the last survey. With regard to advanced prehospital resuscitation interventions, extracorporeal membrane oxygenation (ECMO) was available in five countries (the Netherlands, Germany, Italy, Spain and Sweden), and resuscitative endovascular balloon occlusion of the aorta (REBOA) was available in three countries (Germany, Italy and Norway, no change since previous survey). Both interventions were available in Germany and Italy.

### Cardiac arrest registries

**Fig. 2:**
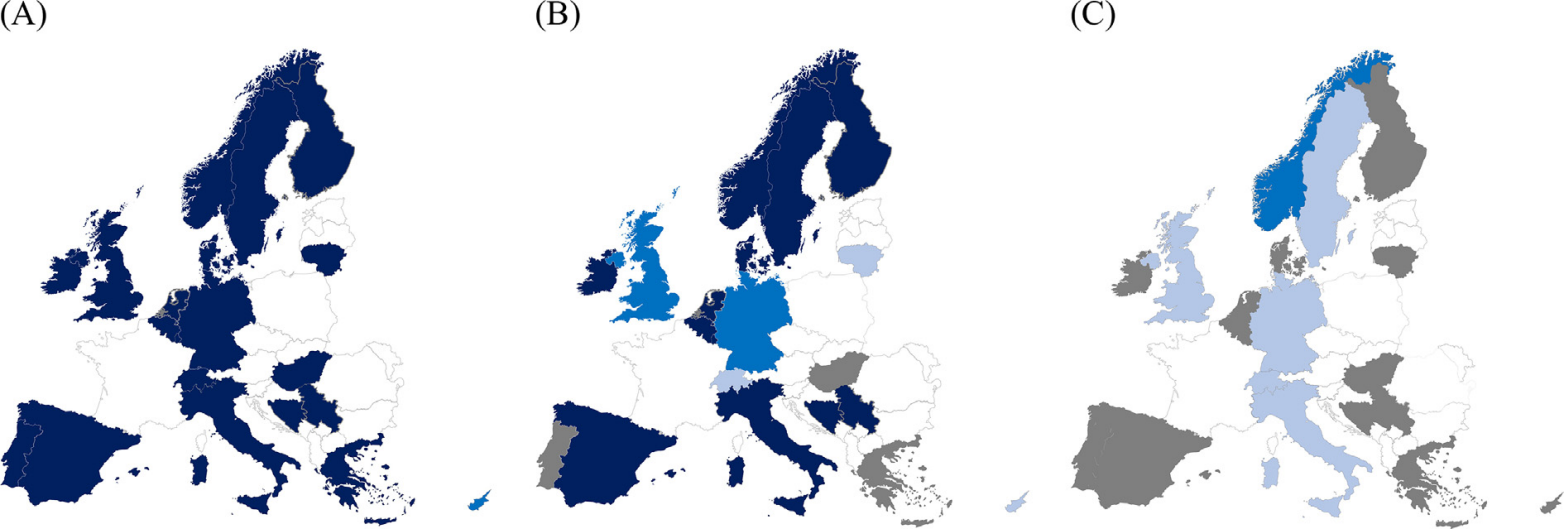
A, B and C: Figure A is ambulance data, figure B is hospital data and figure C is patient reposted quality of life data. Availability of data in out-of-hospital cardiac arrest registries in Europe. Dark brue is availability for over 80 % of the cases, blue is availability of data from 50 to 80 % of cases and light blue is less than 50%. Grey means data is not collected. (For interpretation of the references to colour in this figure legend, the reader is referred to the web version of this article.). (For interpretation of the references to colour in this figure legend, the reader is referred to the web version of this article.)

Twenty-two countries reported having an OHCA registry, with 18 countries having information about EMS confirmed cardiac arrests for all covered areas and four countries having information from some areas (Supplementary table 6). Nine countries reported having a registry with full population coverage, an increase of three countries since the last survey. Thirteen countries reported partial coverage, with five countries reporting no OHCA registry (Albania, Latvia, Poland, Slovakia and Slovenia). Only Denmark, Norway and Sweden had an in-hospital cardiac arrest (IHCA) registry with full population coverage, with seven countries reporting partial coverage (Supplementary table 7). Fifteen countries had no registry data collection for IHCA. Respondents were also asked more detailed questions about the coverage and completeness of variables included in their OHCA (Supplementary table 8) and IHCA registries (Supplementary table 9) Fig. 2.

## Discussion

This survey provides an updated overview of European EMS systems. Understanding the multifaceted way in which EMS organisations are structured is an important part of understanding how EMS systems perform and act globally. This is important from two perspectives: for countries that are at an early stage of EMS development, it is important to have an overview of EMS organisation in other countries and; to ensure European countries have an overview of European EMS systems so that opportunities to learn from each other – and emulate each other – can be identified. Prior to conducting the survey, a search of the literature showed that, while there was a series of studies published in the mid-2000s^15^, ^16^, ^17^, ^18^, ^19^ and mid-2010s,^12^, ^20^, ^21^ there is a lack of recent studies on EMS organisation within Europe and globally. A previous study was conducted by our group, in which 28 countries participated.^8^ However, there was a need to update the survey to obtain information about changes that have occurred within the European EMS organisations in the past five years. Similar questions were posed in this survey, with minor adjustments. When compared to the last survey, it is important to note that two new countries (Bosnia Herzegovina and Latvia) participated, while responses were not received from two other countries (Romania and France) that participated in the previous study.

There have been changes in European EMS organisation since the last survey. While it is obvious that increased availability of AED registers and increased OHCA data collection is a desirable development for any EMS system, the implications of other changes are less clear, as the reasons for these changes are not stated. For example, when compared to the previous survey, five countries reported that less of their personnel were now ALS trained. This suggests that staff with ALS skills now comprise a smaller percentage of the overall EMS workforce in these countries. This may reflect an increase in recruitment of EMS personnel with more basic first aid skills rather than a decrease in the absolute number of personnel with ALS skills. Similarly, there has been an increase in mechanical CPR during transport in many countries since the last survey. This may be because of increased availability of advanced life-saving procedures in hospitals that warrant transport e.g. ECMO or may reflect introduction of mechanical CPR as a safety measure during transport. Our study highlights that there are differences and presents an opportunity for European EMS managers to investigate the implications of these differences with their European EMS peers.

Our survey shows disparities between countries in having access to specialised cardiac arrest centres. Seven out of 27 countries had increased access to specialised cardiac arrest centres, while five reported less access to specialised cardiac arrest centres. The best availability was found in five countries where all residents have access to specialised cardiac arrest centres (Czech Republic, Germany, Luxembourg, the Netherlands and Norway). While there is more research needed with regard to the specific role of cardiac arrest centres, for OHCA patients with a cardiac aetiology, it is important to maximize the availability of 24-hour access to diagnostics and specialised cardiology interventions that minimize the impact of ischaemic injury, and provide acute cardiac care and specialised neuro-prognostication.^22^

Previous research has shown the importance of shorter delays between collapse and the first response from the dispatch centre.^15^ This survey shows a variation in the number of dispatch centres per million inhabitants, varying from 0.4 to 42.2 per million inhabitants and that EMS is usually integrated with dispatch centres. This variation is most likely reflective of how EMS systems are administered e.g. national EMS service provided by health service vs. local EMS service provided by local health authority or council. However, it is unknown how national dispatch centres differ in terms of operations and thus it is problematic to compare the implications of more centralised versus more dispersed dispatch centres between European countries, and it is not possible to comment on which model is ‘best’. What is clear is that there is considerable heterogeneity of EMS dispatch across the continent and investigation of the advantages and disadvantages of both models may be warranted.

Response time is an important factor when it comes to improving the survival of OHCA, to be on the scene as soon as possible.^23^ This survey documents variations in how EMS response time is calculated. The first timestamp used can be any time from when the first call starts at the first answering point to the time the first ambulance is dispatched to the patient. There could easily be a delay of several minutes from the call starting to the ambulance being dispatched, just from the dispatch centre recognising the emergency, identifying the address and then dispatching an ambulance. For the last time stamp used, it is most common to use ambulance arrival at a defined address, but also here there are differences, and several systems use arrival at the patient side.^24^ Within countries, EMS call-response interval is often a key performance metric for EMS and the time stamps used to calculate this metric may be long-standing or prescribed by the EMS regulator. Within countries, it is less important which time stamps are used, but more important that they are reliably used so that any increases or decreases can be nationally tracked and acted upon. When comparing internationally, it is essential that the same time stamps are used by all countries. In the case of OHCA, the consensus-based Utstein reporting template provides detailed description on which timestamps should be used.^25^ While a solution has been found for OHCA, the issue of agreeing time stamps in is not unique to OHCA international research, and other groups have identified this issue and proposed solutions.^26^

A first responder is defined as a volunteer who has been alerted by the dispatch centre in case an OHCA occurs.^27^ In this study, a first responders’ system was available in 17 of 27 countries. However, in our study, we have not investigated whether European countries have implemented systems with voluntary responders, not defined as first responders.

However, most dispatch centres can guide the caller or another bystander to give CPR. High quality chest compressions with early defibrillation are the most important initial treatments for OHCA. However, a previous study has shown that basic CPR skills are lacking, even among healthcare professionals, despite a combination of professional roles such as nurses and physicians.^28^ For this reason, dispatch-assisted CPR is an essential addition to any dispatch centre, and it is encouraging to see that our results show that all countries offer dispatch-assisted bystander CPR, albeit not in all regions.

There are registries with different purposes in the field of cardiac arrest. The three main registries include 1) OHCA 2) IHCA and 3) AED. This survey showed that most European countries have registries of publicly available AEDs. Furthermore, AED databases were reported as available in most of the countries' dispatch centres and more needs to be done to achieve full coverage of all available public defibrillators. There is an increase in the population covered by OHCA registries, but most of the participating countries do not have full coverage. Nine countries cover the entire population, an increase from previous data.^8^ Registers for IHCA are not as common, but Denmark, Norway, and Sweden have full population coverage for IHCA registries.^29^ A previous study describes data quality and its coverage for OHCA and found that 25% of treated OHCAs were not reported.^30^ Globally, more studies on data quality are needed. With regards to the establishment of registers, it should be a routine that the data is continuously reviewed to provide an accurate outcome so that data can be compared between registers.

Our survey indicates that most OHCA registries are good at capturing relevant information for most cases if the information can be collected from the EMS system. However, information from hospitals, and especially information on neurological status and health-related quality of life, is not commonly collected. If registry data is used to describe the incidence and outcome of cardiac arrest, it is important to recognise the limited data available. If reporting to stakeholders in dispatch centres, hospitals or stakeholders involved in recovery and rehabilitation, the data needs to be tailored to their needs, and the data needs to be accessible and available.^23^

### Strengths and imitations

The survey was distributed to national coordinators of the EuReCa studies and contacts in the European Resuscitation council, resulting in only 32 of 44 European countries receiving an invitation. Also, the EuReCa network was not set up to explain the EMS system but is primarily focusing on OHCA management and data collection. While this may introduce selection bias, respondents were assumed to have significant knowledge of their country’s EMS system and to be well placed to provide this information.

The respondents were required to respond on a national level, limiting the ability to capture regional variations within countries. Although response options such as “sometimes” or “in some areas” were available, the overall focus remained on national-level data. Additionally, as the survey was conducted in English, a language not native to most respondents, variations in question interpretation could have affected responses. Differences in EMS terminology and definitions across countries may have further influenced the consistency of the data. Language barriers and different interpretations of definitions might also explain some of the changes from the former survey in 2019 to the current survey however, changes were verified by repeated confirmation of both the results and the changes from the last survey by sharing the final tables with all respondents.

Finally, the study relied on self-reported data without independent verification, and there was variation in how specific EMS metrics, such as response times, were measured across countries. These factors may affect the comparability and reliability of the findings. Despite these limitations, the survey highlights key differences in European EMS systems and raises new research questions. Future studies should aim to explore correlations between EMS characteristics and patient outcomes, particularly survival after OHCA and trauma, while incorporating standardised data collection methods and validation processes.

## Conclusion

The survey shows that there are still differences between EMS systems in Europe. Since the previous study was conducted, there have been improvements for EMS, particularly in OHCA data collection. There is an opportunity for EMS providers to further investigate the implications of these differences, and learn from each other’s practice to optimize EMS provision across Europe.

## Ethical approval

The data in this study consists of publicly available data, and no ethical approval for collection and analysis was needed. An approval was obtained from the data protection officer at Oslo University Hospital for storage and analysis of data.

## Consent for publication

Not applicable.

## Availability of data and materials

The individual responses to the survey are available from the corresponding author on reasonable request. Consent from all involved participants will be sought before sharing.

## Declaration of interest

**Anneli Strömsöe:** Member of the study management team of the European Registry of Cardiac Arrest. Member of the writing group of the chapter on “Epidemiology in Resuscitation” for the ERC guidelines on Resuscitation 2025.

**Ingvild B. M. Tjelmeland:** Daily manager of the Norwegian Cardiac Arrest Registry. Member of the study management team of the European Registry of Cardiac Arrest. Author on the ILCOR Scientific Statement; Cardiac arrest and cardiopulmonary resuscitation: 2024 update of the Utstein Out-of-Hospital Cardiac Arrest Registry Template. Member of the writing group of the chapter on “Epidemiology in Resuscitation” for the ERC guidelines on Resuscitation 2025. First author of the previous paper on EMS systems published in 2020.

**Siobhán Masterson:** Member of the study management team of the European Registry of Cardiac Arrest. Member of the writing group of the chapter on “Epidemiology in Resuscitation” for the ERC guidelines on Resuscitation 2025. Emeritus member of the International Liaison Committee on Resuscitation Basic Life Support Task Force. Coauthor of the previous paper on EMS systems published in 2020. Dr Masterson is a member of the Editorial Board for Resuscitation Plus.

## CRediT authorship contribution statement

**Anneli Strömsöe:** Writing – review & editing, Writing – original draft, Visualization, Project administration, Methodology, Formal analysis, Data curation, Conceptualization. **Ingvild Tjelmeland:** Writing – review & editing, Writing – original draft, Visualization, Project administration, Methodology, Formal analysis, Data curation, Conceptualization. **Siobhan Masterson:** Writing – review & editing, Writing – original draft, Visualization, Validation, Project administration, Methodology, Formal analysis, Data curation, Conceptualization.

## Declaration of generative AI and AI-assisted technologies in the writing process

During the preparation of this work the authors used Chat GPT in order to generate the graphical abstract. After using this tool/service, the authors reviewed and edited the content as needed and take full responsibility for the content of the publication.

## Funding

No funding was received for this study.

## Declaration of competing interest

The authors declare that they have no known competing financial interests or personal relationships that could have appeared to influence the work reported in this paper.
