# Supplementary material for: Emergency medical services, treatment of cardiac arrest patients and cardiac arrest registries in Europe – Update on systems
Source: Resusc Plus. 2025 Apr 16;23:100960. doi: 10.1016/j.resplu.2025.100960 (PMC12135384; doi:10.1016/j.resplu.2025.100960)
Supplement: Supplementary Data 1 [file mmc1.docx]

# Supplementary tables

[Supplementary table 1: Baseline characteristics 2](#_Toc193880295)

[Supplementary table 2: The time stamps used when calculating ambulance response interval. 3](#_Toc193880296)

[Supplementary table 3: Ambulance service characteristics 4](#_Toc193880297)

[Supplementary table 4: Dispatch characteristics 5](#_Toc193880298)

[Supplementary table 5: On scene management of out-of-hospital cardiac arrest 6](#_Toc193880299)

[Supplementary table 6: Cardiac arrest registry coverage and outcome variables for out-of-hospital cardiac arrest 7](#_Toc193880300)

[Supplementary table 7: Cardiac arrest registry coverage and outcome variables for in-hospital cardiac arrest 8](#_Toc193880301)

[Supplementary table 8: Information availability for variables on out-of-hospital cardiac arrest 9](#_Toc193880302)

[Supplementary table 9: Information availability for variables on in-hospital cardiac arrest 10](#_Toc193880303)

# Supplementary table 1: Baseline characteristics

|  | **Population 2023** | **Land area** | **Population per km2** | **Funding** | **"Cardiac Arrest" hospitals** | **Rural areas - median ambulance response time <10 minutes?** | **Urban - median ambulance response time <10 minutes?** |
| --- | --- | --- | --- | --- | --- | --- | --- |
| **Albania** | 2793592 | 28791 | 97 | Public | Yes, some areas | Yes, some areas | Yes, some areas |
| **Austria** | 9104772 | 83882 | 109 | Public | Yes, some areas | Yes, some areas | Yes, all areas |
| **Belgium** | 11742796 | 30667 | 383 | Private and public | Yes, some areas | No | Yes, all areas |
| **Bosnia and Herzegovina** | 3151696 | 51000 | 62 | Public | None | No | Yes, some areas |
| **Cyprus** | 920701 | 9253 | 99 | Public | None | Yes, some areas | Yes, all areas |
| **Czech Republic** | 10827529 | 78871 | 137 | Public | Yes, all areas | No | Yes, all areas |
| **Denmark** | 5932654 | 42925 | 138 | Public | Yes, some areas | Yes, some areas | Yes, all areas |
| **Finland** | 5563970 | 338363 | 16 | Public | Yes, some areas | Yes, some areas | Yes, all areas |
| **Germany** | 84358845 | 357569 | 236 | Public | Yes, all areas | Yes, some areas | Yes, some areas |
| **Greece** | 10413982 | 131694 | 80 | Public | Yes, some areas | Yes, some areas | Yes, some areas |
| **Hungary** | 9599744 | 93012 | 103 | Public | Yes, some areas | Unknown | Unknown |
| **Ireland** | 5271395 | 69947 | 75 | Public | Yes, some areas | Yes, some areas | Yes, some areas |
| **Italy** | 58997201 | 302073 | 195 | Public | Yes, some areas | Yes, some areas | Yes, some areas |
| **Latvia** | 1883008 | 64594 | 30 | Public | Yes, some areas | Yes, some areas | Yes, some areas |
| **Lithuania** | 2857279 | 65284 | 44 | Private and public | Yes, some areas | No | Yes, some areas |
| **Luxembourg** | 660809 | 2595 | 255 | Public | Yes, all areas | Yes, some areas | Yes, some areas |
| **Netherlands** | 17811291 | 37391 | 476 | Private and public | Yes, all areas | Yes, all areas | Yes, all areas |
| **Norway** | 5488984 | 384482 | 14 | Public | Yes, all areas | Yes, some areas | Yes, some areas |
| **Poland** | 36753736 | 311928 | 118 | Private and public | Yes, some areas | Yes, some areas | Yes, some areas |
| **Portugal** | 10516621 | 92226 | 114 | Public | Yes, some areas | Yes, some areas | Yes, some areas |
| **Serbia** | 6797105 | 75106 | 91 | Public | None | No | Yes, some areas |
| **Slovakia** | 5428792 | 49035 | 111 | Private and public | None | Yes, some areas | Yes, all areas |
| **Slovenia** | 2116972 | 20273 | 104 | Public | Yes, some areas | Yes, some areas | Yes, all areas |
| **Spain** | 48085361 | 505983 | 95 | Public | Yes, some areas | Yes, some areas | Yes, all areas |
| **Sweden** | 10521556 | 447424 | 24 | Private and public | Yes, some areas | Yes, some areas | Yes, some areas |
| **Switzerland** | 8815385 | 41287 | 214 | Private and public | Yes, some areas | Yes, some areas | Yes, some areas |
| **United Kingdom** | 66647112 | 244423 | 273 | Public | Yes, some areas | No | Yes, some areas |

Table 1: Baseline characteristics of participating countries. For the country's population official numbers from the EU were used [1]. Grey is no change, blue is more and yellow is less, compared with the previous report. Abbreviations: “Cardiac arrest hospitals” - hospitals capable of providing all of the following post-resuscitation interventions: 24/7 primary PCI, targeted temperature management and neuro-prognostication.

# Supplementary table 2: The time stamps used when calculating ambulance response interval.

|  | **When calculating response interval for the ambulance, what timestamp from the dispatch centre is used?** | **When calculating response interval for the ambulance, what timestamp from the ambulance is used?** |
| --- | --- | --- |
| **Albania** | Time call answered at first answering point | Time of first ambulance resource arrival at defined address (i.e. car stopped) |
| **Austria** | Time call answered in medical/ambulance dispatch centre if this is not the primary answering point | Time of first ambulance resource arrival at defined address (i.e. car stopped) |
| **Belgium** | Time call started at first answering point | Time of first ambulance resource arrival at defined address (i.e. car stopped) |
| **Bosnia and Herzegovina** | Time call started at first answering point | Time of first ambulance resource arrival at defined address (i.e. car stopped) |
| **Cyprus** | Time call answered at first answering point | Time of arrival at patient side |
| **Czechia** | Time call started at first answering point | Time of first ambulance resource arrival at defined address (i.e. car stopped) |
| **Denmark** | Time call started in medical/ambulance dispatch centre if this is not the primary answering point | Other |
| **Finland** | Time call answered at first answering point | Time of arrival at patient side |
| **Germany** | Time call started at first answering point | Time of first ambulance resource arrival at defined address (i.e. car stopped) |
| **Greece** | Time first ambulance resource dispatched to OHCA | Other |
| **Hungary** | Time first ambulance resource dispatched to OHCA | Time of first ambulance resource arrival at defined address (i.e. car stopped) |
| **Ireland** | Time call started in medical/ambulance dispatch centre if this is not the primary answering point | Time of first ambulance resource arrival at defined address (i.e. car stopped) |
| **Italy** | Time call started at first answering point | Time of first ambulance resource arrival at defined address (i.e. car stopped) |
| **Latvia** | Time when call is finished by medical/ambulance dispatche | Time of arrival at patient side |
| **Lithuania** | Time call started at first answering point | Time of arrival at patient side |
| **Luxembourg** | Time call started at first answering point | Time of first ambulance resource arrival at defined address (i.e. car stopped) |
| **Netherlands** | Time call answered in medical/ambulance dispatch centre if this is not the primary answering point | Time of first ambulance resource arrival at defined address (i.e. car stopped) |
| **Norway** | Time call answered at first answering point | Time of first ambulance resource arrival at defined address (i.e. car stopped) |
| **Poland** | Unknown | Time of first ambulance resource arrival at defined address (i.e. car stopped) |
| **Portugal** | Time first ambulance resource dispatched to OHCA | Time of first ambulance resource arrival at defined address (i.e. car stopped) |
| **Serbia** | Time first ambulance resource dispatched to OHCA | Time of first ambulance resource arrival at defined address (i.e. car stopped) |
| **Slovakia** | Time first ambulance resource dispatched to OHCA | Time of first ambulance resource arrival at defined address (i.e. car stopped) |
| **Slovenia** | Time call answered in medical/ambulance dispatch centre if this is not the primary answering point | Time of first ambulance resource arrival at defined address (i.e. car stopped) |
| **Spain** | Time call answered in medical/ambulance dispatch centre if this is not the primary answering point | Time of arrival at patient side |
| **Sweden** | Time call started at first answering point | Time of arrival at patient side |
| **Switzerland** | Time call answered at first answering point | Time of first ambulance resource arrival at defined address (i.e. car stopped) |
| **United Kingdom** | Time call started in medical/ambulance dispatch centre if this is not the primary answering point | Time of first ambulance resource arrival at defined address (i.e. car stopped) |

Supplementary Table 2: The time stamps used when calculating ambulance response interval.

# Supplementary table 3: Ambulance service characteristics

|  | **What is the level of training  of ambulance personnel in your country** | **What is the occupation of the majority of health care providers in the EMS** | **Do physicians provide patient care on scene for OHCA patients as part of EMS** | **ALS trained ambulance personnel can, without the precense of a doctor:** | | | |
| --- | --- | --- | --- | --- | --- | --- | --- |
| **Albania** | All are ALS trained | Emergency physician | Yes, routinely | Secure airways | Intravenous/IO drug therapy | Manual defibrillation | Semi-automatic defibrillation |
| **Austria** | Some are ALS trained | EMT | Yes, routinely | Secure airways | Intravenous/IO drug therapy |  | Semi-automatic defibrillation |
| **Belgium** | Some are ALS trained | Emergency nurse | Yes, routinely | Secure airways | Intravenous/IO drug therapy | Manual defibrillation | Semi-automatic defibrillation |
| **Bosnia and Herzegovina** | Some are ALS trained | Other | Yes, routinely | No | No | No | No |
| **Cyprus** | All are ALS trained | Emergency nurse | No | Secure airways | Intravenous/IO drug therapy | Manual defibrillation | Semi-automatic defibrillation |
| **Czech Republic** | All are ALS trained | Emergency nurse | Yes, routinely | Secure airways | Intravenous/IO drug therapy | Manual defibrillation | Semi-automatic defibrillation |
| **Denmark** | All are ALS trained | Paramedic | Yes, routinely |  | Intravenous/IO drug therapy | Manual defibrillation | Semi-automatic defibrillation |
| **Finland** | Some are ALS trained | EMT | Yes, sometimes | Secure airways | Intravenous/IO drug therapy | Manual defibrillation | Semi-automatic defibrillation |
| **Germany** | All are ALS trained | Paramedic | Yes, routinely | Secure airways | Intravenous/IO drug therapy | Manual defibrillation | Semi-automatic defibrillation |
| **Greece** | Some are ALS trained | Paramedic | Yes, sometimes | No | No | No | Semi-automatic defibrillation |
| **Hungary** | Some are ALS trained | EMT | Yes, routinely | Secure airways | Intravenous/IO drug therapy | Manual defibrillation | Semi-automatic defibrillation |
| **Ireland** | Some are ALS trained | Paramedic | No | Secure airways | Intravenous/IO drug therapy | Manual defibrillation | Semi-automatic defibrillation |
| **Italy** | Some are ALS trained | Other | Yes, routinely | Secure airways | Intravenous/IO drug therapy |  | Semi-automatic defibrillation |
| **Latvia** | All are ALS trained | Other | Yes, routinely | Secure airways | Intravenous/IO drug therapy | Manual defibrillation | Semi-automatic defibrillation |
| **Lithuania** | Some are ALS trained | Emergency nurse | Yes, sometimes | Secure airways | Intravenous/IO drug therapy | Manual defibrillation | Semi-automatic defibrillation |
| **Luxembourg** | Some are ALS trained | EMT | Yes, routinely | Secure airways |  |  | Semi-automatic defibrillation |
| **Netherlands** | All are ALS trained | Emergency nurse | Yes, routinely | Secure airways | Intravenous/IO drug therapy | Manual defibrillation | Semi-automatic defibrillation |
| **Norway** | All are ALS trained | EMT | Yes, sometimes | Secure airways | Intravenous/IO drug therapy | Manual defibrillation | Semi-automatic defibrillation |
| **Poland** | Some are ALS trained | Paramedic | Yes, routinely | Secure airways | Intravenous/IO drug therapy | Manual defibrillation | Semi-automatic defibrillation |
| **Portugal** | Some are ALS trained | EMT | Yes, routinely | Secure airways | Intravenous/IO drug therapy | Manual defibrillation | Semi-automatic defibrillation |
| **Serbia** | Some are ALS trained | Emergency physician | Yes, routinely | No | No | No | No |
| **Slovakia** | Some are ALS trained | Paramedic | Yes, routinely | Secure airways | Intravenous/IO drug therapy | Manual defibrillation | Semi-automatic defibrillation |
| **Slovenia** | Some are ALS trained | Paramedic | Yes, routinely | Secure airways | Intravenous/IO drug therapy | Manual defibrillation | Semi-automatic defibrillation |
| **Spain** | All are ALS trained | Paramedic | Yes, routinely | Secure airways | No | No | Semi-automatic defibrillation |
| **Sweden** | All are ALS trained | Emergency nurse | Yes, sometimes | Secure airways | Intravenous/IO drug therapy | Manual defibrillation | Semi-automatic defibrillation |
| **Switzerland** | All are ALS trained | Paramedic | Yes, sometimes | Secure airways | Intravenous/IO drug therapy | Manual defibrillation | Semi-automatic defibrillation |
| **United Kingdom** | Some are ALS trained | Paramedic | Yes, sometimes | Secure airways | Intravenous/IO drug therapy | Manual defibrillation | Semi-automatic defibrillation |

Supplementary table 3: Ambulance Service Characteristics – Training and Occupation of EMS Ambulance Personnel. The answers are for the entire country which means that the answers “Some” and “Sometimes” indicate this is not implemented in all EMS services in the entire country. Grey is no change, blue is more and yellow is less, compared with the previous report. Abbreviations: EMS – Emergency Medical Services; EMT – Emergency Medical Technician, ALS – Advanced Life Support

# Supplementary table 4: Dispatch characteristics

|  | **Number of ambulance dispatch centres are in your country** | **Dispatch centres per million inhabitants** | **Are the dispatch centres part of the ambulance service?** | **Is a standardised dispatch protocol used in your country?** | **Is dispatch-assisted bystander CPR offered?** | **Dispatch-assisted CPR is:** | **Is a standard protocol for dispatch-assisted bystander CPR used?** | **Are there registries of publicly available AEDs in your country?** | **Is a register or database of publicly accessible AEDs available in your ambulance dispatch centre(s)?** |
| --- | --- | --- | --- | --- | --- | --- | --- | --- | --- |
| **Albania** | 1 | 0,4 | Yes, all | Yes, in all dispatch centres | Yes, in all areas | Compressions only CPR | Yes, in all areas | Yes, in some areas | Yes, in all areas |
| **Austria** | 15 | 1,6 | Yes, some | Yes, in some dispatch centres | Yes, in all areas | Unknown | Yes, in some areas | Yes, in all areas | Yes, in all areas |
| **Belgium** | 10 | 0,9 | Yes, some | Yes, in all dispatch centres | Yes, in all areas | Compressions only CPR | Yes, in all areas | Yes, in some areas | No |
| **Bosnia and Herzegovina** | 133 | 42,2 | Yes, some | Yes, in some dispatch centres | Yes, in some areas | Situation dependent | Yes, in some areas | Yes, in some areas | Yes, in some areas |
| **Cyprus** | 20 | 21,7 | Yes, all | Yes, in all dispatch centres | Yes, in all areas | Compressions only CPR | Yes, in all areas | Yes, in all areas | No |
| **Czech Republic** | 14 | 1,3 | Yes, all | Yes, in some dispatch centres | Yes, in all areas | Compressions only CPR | Yes, in all areas | Yes, in all areas | Yes, in all areas |
| **Denmark** | 5 | 0,8 | Yes, all | Yes, in all dispatch centres | Yes, in all areas | Situation dependent | Yes, in some areas | Yes, in all areas | Yes, in all areas |
| **Finland** | 6 | 1,1 | No | Yes, in all dispatch centres | Yes, in all areas | Situation dependent | Yes, in all areas | Yes, in all areas | No |
| **Germany** | 232 | 2,8 | Yes, all | Yes, in some dispatch centres | Yes, in some areas | Unknown | Yes, in some areas | Yes, in some areas | Yes, in some areas |
| **Greece** | 12 | 1,2 | Yes, all | Yes, in all dispatch centres | Yes, in some areas | Situation dependent | No | Yes, in some areas | Yes, in some areas |
| **Hungary** | 19 | 2,0 | Yes, all | Yes, in all dispatch centres | Yes, in all areas | Compressions only CPR | Yes, in all areas | No | Some |
| **Ireland** | 1 | 0,2 | Yes, all | Yes, in all dispatch centres | Yes, in all areas | Situation dependent | Yes, in all areas | Yes, in all areas | Yes, in all areas |
| **Italy** | 70 | 1,2 | Yes, some | Yes, in some dispatch centres | Yes, in some areas | Situation dependent | Yes, in some areas | Yes, in some areas | Yes, in some areas |
| **Latvia** | 1 | 0,5 | Yes, all | Yes, in all dispatch centres | Yes, in all areas | Situation dependent | Yes, in all areas | Yes, in all areas | Yes, in all areas |
| **Lithuania** | 5 | 1,7 | Yes, all | Yes, in some dispatch centres | Yes, in all areas | Situation dependent | Yes, in some areas | Yes, in some areas | Yes, in some areas |
| **Luxembourg** | 1 | 1,5 | Yes, all | Yes, in all dispatch centres | Yes, in all areas | Compressions only CPR | Yes, in all areas | Yes, in all areas | Yes, in all areas |
| **Netherlands** | 18 | 1,0 | Yes, all | Yes, in all dispatch centres | Yes, in all areas | Compressions only CPR | Yes, in all areas | Yes, in all areas | No |
| **Norway** | 16 | 2,9 | Yes, all | Yes, in all dispatch centres | Yes, in all areas | Situation dependent | Yes, in all areas | Yes, in all areas | Yes, in all areas |
| **Poland** | 17 | 0,5 | Yes, all | Yes, in all dispatch centres | Yes, in all areas | Situation dependent | Yes, in all areas | No |  |
| **Portugal** | 6 | 0,6 | Yes, some | Yes, in all dispatch centres | Yes, in some areas | Compressions only CPR | Yes, in some areas | Yes, in some areas | Yes, in all areas |
| **Serbia** | 140 | 20,6 | Yes, some | No | Yes, in some areas | Situation dependent | No | No |  |
| **Slovakia** | 8 | 1,5 | No | Yes, in all dispatch centres | Yes, in all areas | Compressions only CPR | Yes, in all areas | Yes, in all areas | Yes, in all areas |
| **Slovenia** | 2 | 0,9 | No | Yes, in all dispatch centres | Yes, in all areas | Compressions only CPR | Yes, in all areas | Yes, in some areas | Yes, in some areas |
| **Spain** | 19 | 0,4 | Yes, all | Yes, in some dispatch centres | Yes, in all areas | Situation dependent | Yes, in some areas | Yes, in some areas | Yes, in some areas |
| **Sweden** | 20 | 1,9 | Yes, some | Yes, in all dispatch centres | Yes, in all areas | Situation dependent | Yes, in all areas | Yes, in all areas | Yes, in all areas |
| **Switzerland** | 15 | 1,7 | Yes, some | Yes, in all dispatch centres | Yes, in all areas | Situation dependent | Yes, in all areas | Yes, in all areas | Yes, in all areas |
| **United Kingdom** | 31 | 0,5 | Yes, all | Yes, in all dispatch centres | Yes, in all areas | Situation dependent | Yes, in all areas | Yes, in all areas | Yes, in all areas |

Supplementary table 4: Dispatch Characteristics. The answers are for the entire country which means that the answers “Some” and “Some areas” indicate this is not implemented in all dispatch centres in the entire country. An empty field means no information was given for that specific question. Grey is no change, blue is more and yellow is less, compared with the previous report. Abbreviations: EMS – Emergency Medical Services; AED – Automated External Defibrillator

# Supplementary table 5: On scene management of out-of-hospital cardiac arrest

|  | **Is mechanical CPR used by ambulance personnel?** | **Is mechanical CPR used during transport?** | **Is thrombolysis used in out-of-hospital cardiac arrest?** | **Are defibrillators (manual and/or automated external) available in vehicles dispatched for cardiac arrest?** | **Is transport with ongoing CPR performed?** |
| --- | --- | --- | --- | --- | --- |
| **Albania** | No |  | No | Yes, in all areas | No |
| **Austria** | Yes, in some areas | Yes, in some areas | Yes, in all areas | Yes, in all areas | Yes, in all areas |
| **Belgium** | No | Yes, in all areas | No | Yes, in all areas | Yes, in all areas |
| **Bosnia and Herzegovina** | Yes, in some areas |  | Yes, in some areas | Yes, in some areas | Unknown |
| **Cyprus** | Yes, in all areas | Yes, in all areas | No | Yes, in all areas | Yes, in all areas |
| **Czech Republic** | Yes, in all areas | Yes, in all areas | Yes, in some areas | Yes, in all areas | Yes, in some areas |
| **Denmark** | Yes, in some areas | Yes, in all areas | No | Yes, in all areas | Yes, in all areas |
| **Finland** | Yes, in some areas | Yes, in some areas | Yes, in some areas | Yes, in all areas | Yes, in some areas |
| **Germany** | Yes, in some areas | Yes, in some areas | Yes, in some areas | Yes, in all areas | Yes, in some areas |
| **Greece** | Yes, in some areas | Yes, in some areas | No | Yes, in all areas | Yes, in some areas |
| **Hungary** | Yes, in all areas | Yes, in all areas | No | Yes, in all areas | Yes, in all areas |
| **Ireland** | Yes, in all areas | Yes, in some areas | No | Yes, in all areas | Yes, in some areas |
| **Italy** | Yes, in some areas | Yes, in some areas | Yes, in some areas | Yes, in all areas | Yes, in some areas |
| **Latvia** | Yes, in some areas | Yes, in some areas | Yes, in some areas | Yes, in all areas | Yes, in some areas |
| **Lithuania** | Yes, in some areas | Yes, in some areas | No | Yes, in all areas | Yes, in some areas |
| **Luxembourg** | Yes, in all areas | Yes, in all areas | Yes, in all areas | Yes, in all areas | Yes, in all areas |
| **Netherlands** | Yes, in some areas | Yes, in some areas | Yes, in all areas | Yes, in all areas | Yes, in all areas |
| **Norway** | Yes, in some areas | Yes, in all areas | Yes, in some areas | Yes, in all areas | Yes, in all areas |
| **Poland** | Yes, in some areas | Yes, in some areas | Unknown | Yes, in all areas | Yes, in some areas |
| **Portugal** | Yes, in some areas | Yes, in all areas | Yes, in some areas | Yes, in all areas | Yes, in all areas |
| **Serbia** | No |  | No | Yes, in all areas | No |
| **Slovakia** | Yes, in some areas | Yes, in some areas | Yes, in some areas | Yes, in all areas | Yes, in all areas |
| **Slovenia** | Yes, in some areas | Yes, in some areas | No | Yes, in all areas | Yes, in some areas |
| **Spain** | Yes, in some areas | Yes, in some areas | Yes, in some areas | Yes, in all areas | Yes, in some areas |
| **Sweden** | Yes, in some areas | Yes, in some areas | Yes, in some areas | Yes, in all areas | Yes, in all areas |
| **Switzerland** | Yes, in some areas | Yes, in some areas | Unknown | Yes, in all areas | Yes, in all areas |
| **United Kingdom** | Yes, in all areas | Yes, in some areas | Yes, in some areas | Yes, in all areas | Yes, in all areas |

Supplementary table 5: On-scene management of out-of-hospital cardiac arrest by emergency medical personnel. Abbreviations: EMS – Emergency Medical Services; OHCA – Out-of-Hospital Cardiac Arrest. Grey is no change, blue is more and yellow is less, compared with the previous report.

# Supplementary table 6: Cardiac arrest registry coverage and outcome variables for out-of-hospital cardiac arrest

| **Country** | **EMS confirmed cardiac arrest** | **EMS treated cardiac arrests** | **Patients defibrillated by AED before EMS arrival and puls when EMS arrives** | **Any ROSC** | **Sustained ROSC** | **Status on hospital arrival** | **Alive at hospital discharge** | **Alive at 30 days** | **Alive at 1 year** | **Neurological status at discharge** | **Neurological status 3-6 months post arrest** | **Neurological status 1 year post arrest** | **HRQoL at 3 months** | **HRQoL 1 year after cardiac arrest** |
| --- | --- | --- | --- | --- | --- | --- | --- | --- | --- | --- | --- | --- | --- | --- |
| **Austria** |  |  |  |  |  |  |  |  |  |  |  |  |  |  |
| **Belgium** |  |  |  |  |  |  |  |  |  |  |  |  |  |  |
| **Bosnia and Herzegovina** |  |  |  |  |  |  |  |  |  |  |  |  |  |  |
| **Cyprus** |  |  |  |  |  |  |  |  |  |  |  |  |  |  |
| **Czech Republic** |  |  |  |  |  |  |  |  |  |  |  |  |  |  |
| **Danmark** |  |  |  |  |  |  |  |  |  |  |  |  |  |  |
| **Finland** |  |  |  |  |  |  |  |  |  |  |  |  |  |  |
| **Germany** |  |  |  |  |  |  |  |  |  |  |  |  |  |  |
| **Greece** |  |  |  |  |  |  |  |  |  |  |  |  |  |  |
| **Hungary** |  |  |  |  |  |  |  |  |  |  |  |  |  |  |
| **Ireland** |  |  |  |  |  |  |  |  |  |  |  |  |  |  |
| **Italia** |  |  |  |  |  |  |  |  |  |  |  |  |  |  |
| **Lithuania** |  |  |  |  |  |  |  |  |  |  |  |  |  |  |
| **Luxembourg** |  |  |  |  |  |  |  |  |  |  |  |  |  |  |
| **Netherlands** |  |  |  |  |  |  |  |  |  |  |  |  |  |  |
| **Norway** |  |  |  |  |  |  |  |  |  |  |  |  |  |  |
| **Portugal** |  |  |  |  |  |  |  |  |  |  |  |  |  |  |
| **Serbia** |  |  |  |  |  |  |  |  |  |  |  |  |  |  |
| **Spain** |  |  |  |  |  |  |  |  |  |  |  |  |  |  |
| **Sweden** |  |  |  |  |  |  |  |  |  |  |  |  |  |  |
| **Switzerland** |  |  |  |  |  |  |  |  |  |  |  |  |  |  |
| **United Kingdom** |  |  |  |  |  |  |  |  |  |  |  |  |  |  |

Supplementary table 6: Cardiac arrest registry coverage and outcome variables collected in the out-of-hospital cardiac arrest registries. Dark blue is all areas in the catchment area of the registry, light blue is some areas in the catchment area of the registry, yellow is not collected, and grey is unknown. Abbreviations: EMS – Emergency Medical Services, ROSC – Return of Spontaneous Circulation, AED – Automated External Defibrillator, HRQoL – Health Related Quality of Life.

# Supplementary table 7: Cardiac arrest registry coverage and outcome variables for in-hospital cardiac arrest

| **Country** | **Date of hospital admissin** | **Event location** | **Event withessed** | **Resuscirtation team called** | **Any CPR or defibrillation** | **Any ROSC** | **Alive at hospital discharge** | **Alive at 30 days** | **Alive at 1 year** | **Neurological status at discharge** | **Neurological status 3-6 months post arrest** | **Neurological status 1 year post arrest** | **HRQoL at 3 months** | **HRQoL 1 year after cardiac arrest** |
| --- | --- | --- | --- | --- | --- | --- | --- | --- | --- | --- | --- | --- | --- | --- |
| **Austria** |  |  |  |  |  |  |  |  |  |  |  |  |  |  |
| **Cyprus** |  |  |  |  |  |  |  |  |  |  |  |  |  |  |
| **Danmark** |  |  |  |  |  |  |  |  |  |  |  |  |  |  |
| **Germany** |  |  |  |  |  |  |  |  |  |  |  |  |  |  |
| **Greece** |  |  |  |  |  |  |  |  |  |  |  |  |  |  |
| **Italia** |  |  |  |  |  |  |  |  |  |  |  |  |  |  |
| **Norway** |  |  |  |  |  |  |  |  |  |  |  |  |  |  |
| **Sweden** |  |  |  |  |  |  |  |  |  |  |  |  |  |  |
| **Switzerland** |  |  |  |  |  |  |  |  |  |  |  |  |  |  |
| **UK** |  |  |  |  |  |  |  |  |  |  |  |  |  |  |

Supplementary Table 2: Cardiac arrest registry coverage and outcome variables collected in in-hospital cardiac arrest registries. Dark blue is all areas in the catchment area of the registry, light blue is some areas in the catchment area of the registry, yellow is not collected, and grey is unknown. Abbreviations: ROSC – Return of Spontaneous Circulation, AED – Automated External Defibrillator, HRQoL – Health Related Quality of Life

# Supplementary table 8: Information availability for variables on out-of-hospital cardiac arrest

|  | **EMS confirmed cardiac arrest** | **EMS treated cardiac arrests** | **Patients defibrillated by AED before EMS arrival and puls when EMS arrives** | **Any ROSC** | **Sustained ROSC** | **Status on hospital arrival** | **Alive at hospital discharge** | **Alive at 30 days** | **Alive at 1 year** | **Neurological status at discharge** | **Neurological status 3-6 months after discharge** | **Neurological status 1 year after discharge** | **HRQoL 3-6 months after cardiac arrest** | **HRQoL 1 year after cardiac arrest** |
| --- | --- | --- | --- | --- | --- | --- | --- | --- | --- | --- | --- | --- | --- | --- |
| **Austria** |  |  |  |  |  |  |  |  |  |  |  |  |  |  |
| **Belgium** |  |  |  |  |  |  |  |  |  |  |  |  |  |  |
| **Bosnia and Herzegovina** |  |  |  |  |  |  |  |  |  |  |  |  |  |  |
| **Cyprus** |  |  |  |  |  |  |  |  |  |  |  |  |  |  |
| **Czech Republic** |  |  |  |  |  |  |  |  |  |  |  |  |  |  |
| **Danmark** |  |  |  |  |  |  |  |  |  |  |  |  |  |  |
| **Finland** |  |  |  |  |  |  |  |  |  |  |  |  |  |  |
| **Germany** |  |  |  |  |  |  |  |  |  |  |  |  |  |  |
| **Greece** |  |  |  |  |  |  |  |  |  |  |  |  |  |  |
| **Hungary** |  |  |  |  |  |  |  |  |  |  |  |  |  |  |
| **Ireland** |  |  |  |  |  |  |  |  |  |  |  |  |  |  |
| **Italia** |  |  |  |  |  |  |  |  |  |  |  |  |  |  |
| **Lithuania** |  |  |  |  |  |  |  |  |  |  |  |  |  |  |
| **Luxembourg** |  |  |  |  |  |  |  |  |  |  |  |  |  |  |
| **Netherlands** |  |  |  |  |  |  |  |  |  |  |  |  |  |  |
| **Norway** |  |  |  |  |  |  |  |  |  |  |  |  |  |  |
| **Portugal** |  |  |  |  |  |  |  |  |  |  |  |  |  |  |
| **Serbia** |  |  |  |  |  |  |  |  |  |  |  |  |  |  |
| **Spain** |  |  |  |  |  |  |  |  |  |  |  |  |  |  |
| **Sweden** |  |  |  |  |  |  |  |  |  |  |  |  |  |  |
| **Switzerland** |  |  |  |  |  |  |  |  |  |  |  |  |  |  |
| **United Kingdom** |  |  |  |  |  |  |  |  |  |  |  |  |  |  |

Table 6: The rate of eligible patients in the out-of-hospital cardiac arrest registry for whom information is documented. Dark blue is over 80 %, blue is 50 – 80 %, light blue is under 50 %, yellow is none, grey is unknown. Abbreviations: EMS – Emergency Medical Services, ROSC – Return of Spontaneous Circulation, AED – Automated External Defibrillator, HRQoL – Health Related Quality of Life.

# Supplementary table 9: Information availability for variables on in-hospital cardiac arrest

| **Country** | **Date of hospital admissin** | **Event location** | **Event withessed** | **Resuscirtation team called** | **Any CPR or defibrillation** | **Any ROSC** | **Alive at hospital discharge** | **Alive at 30 days** | **Alive at 1 year** | **Neurological status at discharge** | **Neurological status 3-6 months post arrest** | **Neurological status 1 year post arrest** | **HRQoL at 3 months** | **HRQoL 1 year after cardiac arrest** |
| --- | --- | --- | --- | --- | --- | --- | --- | --- | --- | --- | --- | --- | --- | --- |
| **Austria** |  |  |  |  |  |  |  |  |  |  |  |  |  |  |
| **Cyprus** |  |  |  |  |  |  |  |  |  |  |  |  |  |  |
| **Danmark** |  |  |  |  |  |  |  |  |  |  |  |  |  |  |
| **Germany** |  |  |  |  |  |  |  |  |  |  |  |  |  |  |
| **Greece** |  |  |  |  |  |  |  |  |  |  |  |  |  |  |
| **Italia** |  |  |  |  |  |  |  |  |  |  |  |  |  |  |
| **Norway** |  |  |  |  |  |  |  |  |  |  |  |  |  |  |
| **Sweden** |  |  |  |  |  |  |  |  |  |  |  |  |  |  |
| **Switzerland** |  |  |  |  |  |  |  |  |  |  |  |  |  |  |
| **UK** |  |  |  |  |  |  |  |  |  |  |  |  |  |  |

Supplementary Table 3: The rate of eligible patients in the in-hospital cardiac arrest registry for whom information is documented. Dark blue is all areas in the catchment area of the registry, light blue is some areas in the catchment area of the registry, yellow is not collected, and grey is unknown. Abbreviations: ROSC – Return of Spontaneous Circulation, AED – Automated External Defibrillator, HRQoL – Health Related Quality of Life.

Literature list

1. Eurostat. *Population on 1 January by age group, sex and country of birth*. 2024 04/11/2024 [cited 2025 06.01].
